# Accurate Segmentation of Overlapping Cervical Cells Using an Optimized Deep Learning Framework for Cytology Screening

**DOI:** 10.3390/diagnostics16142240

**Published:** 2026-07-17

**Authors:** Amal A. Alzu’bi, Mohammad Khatatbeh, Wan Azani Mustafa, Norhayati Mohd Zain, Hiam Alquran, Alia Al-Mohtaseb, Khaled Z. Alawneh, Mohammad Fawaeer, Bara’a Fawaeer, Shatha Salameh, Ahmad Alhussain

**Affiliations:** 1 Department of Computer Information Systems, Jordan University of Science and Technology, P.O. Box 3030, Irbid 22110, Jordan; aazoubi9@just.edu.jo (A.A.A.); mykhatatbeh23@cit.just.edu.jo (M.K.); 2 Advanced Computing (AdvCOMP), Centre of Excellence, Universiti Malaysia Perlis (UniMAP), Pauh Putra Campus, Arau 02600, Perlis, Malaysia; 3 Medical Imaging Department, School of Integrative Medicine & Life Sciences, KPJ Healthcare University, Nilai 71800, Negeri Sembilan, Malaysia; 4 Department of Biomedical Systems and Informatics Engineering, Yarmouk University, P.O. Box 566, Irbid 21163, Jordan; heyam.q@yu.edu.jo; 5 Department of Pathology, Jordan University of Science and Technology, P.O. Box 3030, Irbid 22110, Jordan; ahmohtaseb@just.edu.jo; 6 Department of Radiology and Nuclear Medicine, Jordan University of Science and Technology, P.O. Box 3030, Irbid 22110, Jordan; kzalawneh0@just.edu.jo; 7 Public Health & Preventive Medicine Division, Directorate of the Royal Medical Services, P.O. Box 855005, Amman 11855, Jordan; mohfawaeer@gmail.com; 8 Hematology and Blood Banking, Ministry of Health, Princess Basma Teaching Hospital, Irbid 21110, Jordan; baraafawaeer@gmail.com; 9 Department of Obstetrics and Gynecology, King Abdullah University Hospital, Jordan University of Science and Technology, P.O. Box 3030, Irbid 22110, Jordan; stsalameh211@gmail.com; 10 Department of General Surgery, King Abdullah University Hospital, P.O. Box 3030, Irbid 22110, Jordan; justunimed@gmail.com

**Keywords:** cervical cancer, pap smear, mask R-CNN, instance segmentation, overlapping cells, medical image analysis, computer-aided screening, small-cell detection

## Abstract

**Background**: Cervical cancer remains one of the leading causes of cancer-related morbidity among women worldwide. The Papanicolaou (Pap) smear is widely used for early detection; however, its manual interpretation is time-consuming, requires substantial expertise, and is often affected by inter-observer variability, particularly in cases with dense and overlapping cells. **Methods**: This study developed an optimized deep learning framework for cervical cell instance segmentation, specifically targeting the separation of overlapping cells in Pap smear images. The proposed framework was based on Mask R-CNN with a ResNet-50 backbone and Feature Pyramid Network. A public development dataset of 460 cervical smear images was used for model development and internal evaluation, while an independent 210-image dataset collected from King Abdullah University Hospital was reserved as an external held-out clinical assessment set. To improve small-cell detection and mask refinement, the Region Proposal Network was adapted using biologically informed anchor scales (16, 32, 64, 128, 256), followed by soft calibration-based post-processing. **Results**: The proposed framework achieved an AP50 of 89.90% and a Mask IoU of 90.44%. Small-cell performance, measured using APs under the COCO small-object convention, reached 22.80%, while APm and APl reached 68.45% and 81.42%, respectively. Clinical cell-count evaluation on the independent KAUH held-out set was performed using the mean count of five physicians as the reference standard and achieved an overall clinical detection accuracy of 93.00%. **Conclusions**: The optimized Mask R-CNN framework improved the detection and separation of overlapping cervical cells in Pap smear images and may serve as a supportive tool for cytopathology workflows. The results suggest that biologically informed anchor optimization and soft calibration can improve cell-level instance segmentation, particularly for small and overlapping cells. Further validation on larger multi-center datasets remains necessary before routine clinical deployment.

## 1. Introduction

Cervical cancer remains a major global health challenge and continues to be one of the leading causes of cancer-related mortality among women worldwide, particularly in low- and middle-income countries [1]. Early detection through the Papanicolaou (Pap) smear has significantly reduced the incidence of precancerous lesions and improved patient outcomes. However, cytological assessment of Pap smear slides is inherently labor-intensive and requires highly trained specialists to examine large numbers of samples. This process is often affected by fatigue, inter-observer variability, and diagnostic inconsistencies, which may compromise screening accuracy [2].

One of the most critical challenges in cervical cytology is the presence of overlapping cells. In many cases, cervical cells appear in dense clusters with complex spatial arrangements, making them difficult to interpret and accurately classify. From a histological perspective, cell intercalation refers to the partial overlap of adjacent squamous epithelial cells, often resulting from sample collection, slide preparation, or fixation processes. This issue is further exacerbated by the presence of mucus, blood contamination, or inflammatory debris, all of which can obscure cellular boundaries. Such conditions reduce slide interpretability and may increase the risk of missed abnormal cells during manual screening, as abnormal cells may remain hidden within overlapping regions.

Over the years, automated cervical cell segmentation has evolved from traditional image-processing techniques to more advanced computational approaches. Early methods relied on intensity thresholding and mathematical modeling. For example, Lu et al. introduced level-set-based approaches for separating overlapping cells, later improving performance through joint optimization strategies [3]. While mathematically robust, these methods are computationally demanding and highly sensitive to initialization, particularly in images with low cytoplasmic contrast.

Other approaches have attempted to combine clustering and morphological operations. Chankong et al. [4] integrated fuzzy C-means clustering with the Watershed algorithm to segment cervical cells. Although effective in relatively simple scenarios, Watershed-based methods tend to produce over-segmentation in densely packed cellular regions. Similarly, Nosrati and Hamarneh [5] proposed a variational framework incorporating star-shape priors; however, such assumptions limit performance when dealing with irregular and non-convex cell morphologies commonly observed in pathological samples.

Despite these advancements, conventional Pap smear analysis remains largely dependent on manual interpretation, which is time-consuming, subjective, and prone to variability [6,7]. To mitigate these limitations, computer-aided diagnostic (CAD) systems were introduced, initially relying on classical machine-learning techniques [8]. More recently, the emergence of deep learning has transformed medical image analysis, with models demonstrating strong performance in object detection and instance segmentation tasks [9,10].

Nevertheless, cervical cytology presents unique challenges that are not fully addressed by standard deep learning models. In particular, dense cellular clusters, cytoplasmic interference, and the presence of small or highly overlapped cells can significantly degrade detection performance. Even widely adopted architectures such as Mask R-CNN may struggle under these conditions, often failing to detect smaller cells or accurately delineate overlapping boundaries [11]. While post-processing techniques, including the Watershed algorithm, are commonly applied to refine segmentation outputs, they can introduce artifacts such as cell fragmentation or distortion of morphological features [12]. These limitations underscore the need for structurally adapted deep learning frameworks tailored specifically to the complexities of cervical smear images.

To address these challenges, this study proposes an optimized Mask R-CNN-based framework for accurate detection and instance segmentation of overlapping cervical cells. The proposed approach incorporates biologically informed anchor optimization and calibration strategies to enhance detection sensitivity across cells of varying sizes while preserving morphological integrity. In addition, targeted post-processing is applied to refine segmentation quality. Through systematic evaluation, this framework aims to improve diagnostic reliability and bridge the gap between advanced deep learning techniques and practical cytological applications, supporting its role as a potential second reader in cervical cancer screening.

The main contribution of this study is the development of an optimized Mask R-CNN framework for cervical cell instance segmentation in dense and overlapping Pap smear images. The proposed framework incorporates three key components: first, a ResNet-50-FPN-based Mask R-CNN architecture adapted for multi-scale cervical-cell representation; second, biologically informed anchor optimization using anchor scales (16, 32, 64, 128, 256) to improve the detection of small and partially overlapping cells; and third, a soft calibration post-processing strategy designed to refine predicted masks while preserving cellular morphology. In addition to computational evaluation on the annotated development dataset, the framework was further assessed on an independent KAUH held-out dataset using clinical cell-count agreement against the mean count of five physicians. These additions aim to improve small-cell sensitivity, reduce mask fragmentation, and support the potential use of the model as an auxiliary tool in cervical cytology screening workflows.

## 2. Related Work

Cervical cancer has historically been one of the leading causes of cancer-related mortality among women worldwide prior to the widespread implementation of cytological screening programs [2,3]. The disease originates in the epithelial cells lining the cervix and progresses gradually through precancerous stages, making early detection crucial for effective treatment and improved survival outcomes.

Several traditional image-processing approaches have been proposed to address the segmentation of overlapping cervical cells. Lu et al. introduced level-set-based formulations for separating overlapping nuclei and cytoplasm [2], later improving the method through joint optimization of multiple level-set functions. Although mathematically robust, these methods are computationally expensive and highly sensitive to initialization, particularly in cases where cytoplasmic contrast is weak.

Chankong et al. proposed a hybrid framework combining fuzzy C-means clustering with the Watershed algorithm for segmentation and classification [4]. While effective for relatively simple overlapping cases, Watershed-based techniques inherently suffer from over-segmentation when applied to dense and complex cellular clusters. Similarly, Nosrati and Hamarneh introduced a variational method incorporating star-shape priors to guide segmentation [5]. However, such geometric assumptions restrict applicability to irregular and non-convex cell morphologies frequently encountered in real clinical samples.

Previous studies have demonstrated that accurate segmentation of overlapping cervical-cell clumps is a fundamental prerequisite for reliable automated cervical cytology analysis [13]. With the rapid advancement of deep learning, convolutional neural networks (CNNs) have become central to medical image analysis [14]. CNN-based methods have been applied to cervical cytology using super pixel generation and hierarchical feature extraction [15]. However, many early CNN-based segmentation approaches failed to resolve severe intercellular overlap adequately. They could not reliably assign pixels to individual cells, which is essential for accurate cell counting and diagnostic assessment [16].

Recent reviews have shown that deep learning-based cervical cytology analysis has rapidly expanded from single-cell classification toward detection, segmentation, and whole-slide image analysis. Jiang et al. reviewed recent studies in this field and emphasized the importance of integrating cytological domain knowledge into computer-aided diagnostic systems. Similarly, Fang et al. summarized recent developments in cervical cell image analysis and highlighted that segmentation remains a central challenge, particularly in images with complex morphology, overlapping cells, and heterogeneous staining conditions [14,16].

He et al. introduced the Mask R-CNN architecture, which significantly advanced instance segmentation by integrating region proposal networks with pixel-wise mask prediction [11]. Subsequent studies adapted Mask R-CNN for cervical cytology applications. Wan et al. and Allehaibi et al. demonstrated its effectiveness in segmentation and classification tasks but reported persistent challenges in separating densely overlapping nuclei [17,18]. Zhou et al. proposed the Instance Relation Network to model pixel-level relationships in overlapping regions; however, performance degradation was observed in highly clustered scenarios [19]. More recently, Zhang et al. integrated a PointRend module into Mask R-CNN to improve boundary refinement and geometric accuracy [20].

Despite these improvements, many advanced frameworks involve substantial computational complexity and long processing times, limiting their direct clinical applicability. In contrast, the proposed framework focuses on optimizing core structural components of the Mask R-CNN architecture and introducing a lightweight morphological calibration step for post-processing [21].

This approach aims to balance segmentation accuracy with computational efficiency, making it more suitable for real-world hospital deployment.

## 3. Materials and Methods

This section describes the stages of the proposed framework, beginning with data acquisition and preparation, followed by image enhancement, dynamic augmentation, model optimization, post-processing, and evaluation.

### 3.1. Dataset Acquisition and Partitioning

This study used a public cervical cytology development image pool assembled from the Herlev benchmark dataset [22] and the SIPaKMeD dataset [23], in addition to an independent private held-out dataset collected from King Abdullah University Hospital (KAUH), Jordan. The study design consisted of a public development pool and a completely separate KAUH external held-out dataset for clinical assessment.

The public development pool included 460 Pap smear images. Within this pool, 346 images had pixel-level COCO-format instance annotations and were used for computational model training, validation, and internal testing. These annotated images were split into 242 training images, 52 validation images, and 52 internal testing images. The remaining 114 public images were retained for image-level inspection and qualitative inference assessment only. They were not included in COCO-style AP50, APs, APm, APl, or Mask IoU calculations because pixel-level instance masks were not available.

The active annotated COCO subset contained 8860 annotated cervical-cell instances. The training subset included 6311 annotated cell instances, the validation subset included 1222 instances, and the internal testing subset included 1327 instances. This partitioning enabled computational training and internal mask-based evaluation using fully annotated images.

In addition, an independent private dataset of 210 Pap smear images was collected from King Abdullah University Hospital. This KAUH dataset was kept completely separate from model training, validation, and internal testing. Because the KAUH images did not include pixel-level instance masks, they were not used for AP50, APs, APm, APl, or Mask IoU computation. Instead, they were used only for external clinical cell-count agreement assessment against physician-derived reference counts. Table 1 summarizes the dataset components, annotation status, and evaluation roles used in this study.

To further clarify the distribution of cervical-cell instance sizes within the annotated subset, cell instances were categorized using the COCO object-size convention after preprocessing and resizing. Instances with a mask area < 32^2^ pixels were considered small, instances with an area between 32^2^ and 96^2^ pixels were considered medium, and instances with an area ≥ 96^2^ pixels were considered large. Table 2 summarizes the distribution of cervical-cell instance sizes across the annotated training, validation, and internal testing subsets.

This distribution shows that small cervical-cell instances represented 7.49%, 7.20%, and 6.71% of the training, validation, and internal testing subsets, respectively, supporting the need for smaller anchor scales during Region Proposal Network optimization.

### 3.2. Image Enhancement and Standardization

One limitation encountered in cervical smear images was the high contrast variation caused by staining type, staining protocols, and sample fixation methods. Therefore, before initiating the enhancement process, the images were preprocessed to reduce staining-related contrast variations and common distortions observed in cervical smear images.

**Figure 1:**
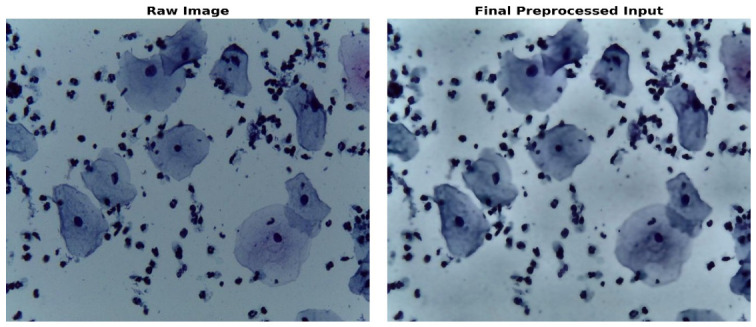
Image enhancement and standardization pipeline. (**Left**): Raw cervical smear image. (**Right**): Final preprocessed image after contrast enhancement and bilateral filtering.

First, a high-frequency bilateral filter was applied to suppress noise while preserving edge discontinuities along cell membranes, thereby maintaining cell boundary integrity within their original limits. The next stage involved contrast-limited adjustment, which is a crucial step for addressing local contrast variations and improving the visibility of cytoplasmic regions and overlapping structures, regardless of the original illumination conditions of the slide. The final step involved standardizing all images to a fixed resolution of 1024 × 1024 pixels. Figure 1 illustrates the image enhancement and standardization pipeline, showing the raw cervical smear image and the final preprocessed image after contrast enhancement and bilateral filtering.

### 3.3. Preprocessing and On-the-Fly Augmentation

Because the development image pool was relatively limited for training a deep learning-based instance segmentation model, preprocessing and on-the-fly augmentation were applied to improve training stability and reduce overfitting. All input images first underwent standardization to reduce variability related to image format, illumination, contrast, and background noise. Image channels were checked and converted to a standard three-channel format when necessary. Bilateral filtering was then applied to reduce local noise while preserving visible cellular boundaries. Contrast enhancement was performed using contrast-limited adaptive histogram equalization (CLAHE), which improved the visibility of nuclei and cytoplasmic structures without excessively amplifying background artifacts.

**Figure 2:**
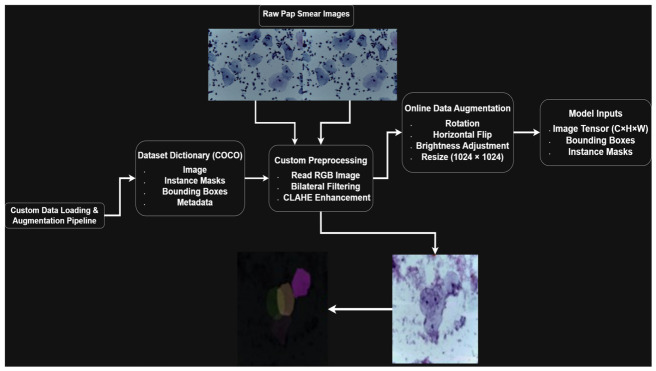
Custom preprocessing and on-the-fly augmentation pipeline applied during model training. The pipeline standardized image channels, reduced local noise, enhanced contrast, and applied dynamic geometric and photometric transformations.

The augmentation pipeline was implemented using the Albumentations library and applied dynamically during training. Unlike static augmentation, which generates a fixed augmented dataset before training, the on-the-fly strategy randomly transforms images during each training cycle. This exposes the model to different transformed versions of the same cervical cells across epochs and encourages the learning of more robust morphological features. The complete preprocessing and on-the-fly augmentation pipeline used during model training is illustrated in Figure 2.

**Figure 3:**
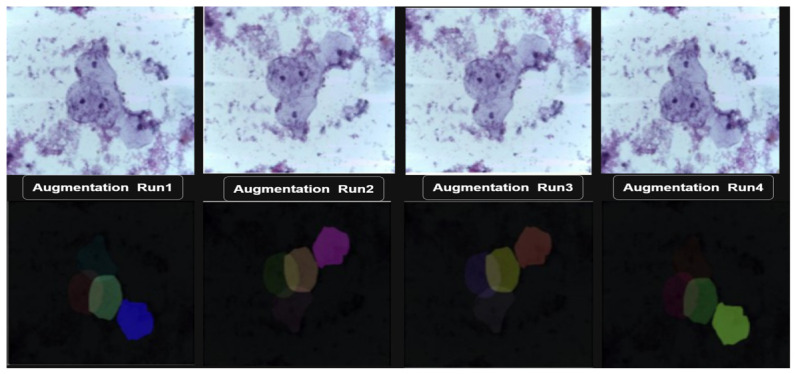
Representative geometric augmentation examples, including random flipping, limited-angle rotation, shifting, scaling, resizing, and padding. The different colors are used only for visualization to distinguish individual annotated cell instances and do not indicate different cell classes. These transformations were applied to improve model robustness to spatial variation in Pap smear images.

**Figure 4:**
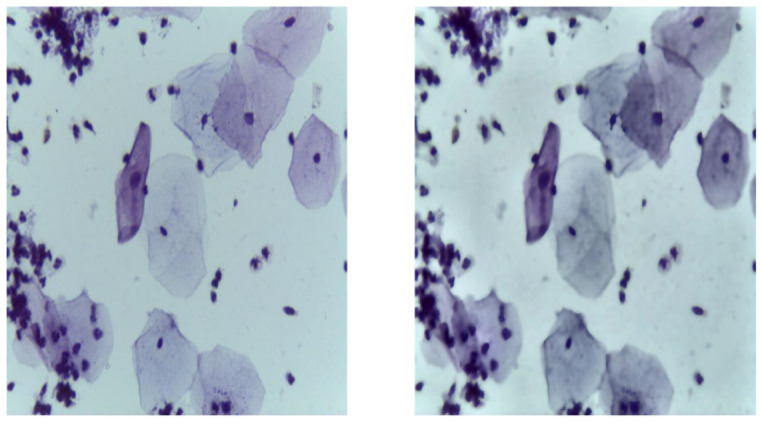
Optical augmentation through random brightness and contrast adjustment. Photometric augmentation was used to simulate staining and illumination variability commonly observed in cervical cytology images.

The augmentation pipeline included both geometric and photometric transformations. Geometric augmentation included random flipping, limited-angle rotation, shifting, scaling, resizing, and padding to standardize the input dimensions. These transformations were used to improve model tolerance to variations in cell orientation and spatial position. Photometric augmentation included random brightness and contrast adjustments and mild noise perturbation to simulate differences in staining intensity, illumination, and slide-preparation conditions. Representative examples of the preprocessing and augmentation pipeline are shown in Figure 3 and Figure 4.

Although on-the-fly augmentation increased the visual diversity of the training data and reduced the risk of overfitting, it was not considered a substitute for larger real-world multi-center datasets. Therefore, the potential effect of limited dataset size and domain shift across centers, scanners, and staining protocols is further discussed in the Section 5 and Section 6.

### 3.4. Optimized Mask R-CNN Architecture

**Figure 5:**
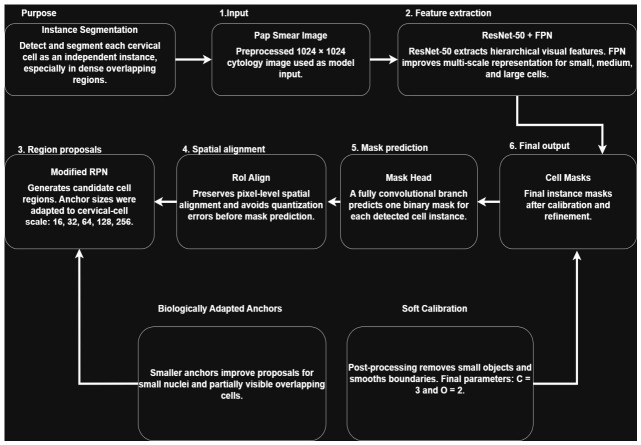
Optimized Mask R-CNN architecture with a ResNet-50 backbone, Feature Pyramid Network, and modified Region Proposal Network. The RPN uses biologically informed anchor scales (16, 32, 64, 128, 256) to improve proposal generation for small and overlapping cervical-cell instances, followed by RoI Align, classification, bounding-box regression, mask prediction, and soft calibration-based mask refinement.

The core of the proposed framework is an optimized Mask R-CNN architecture built upon a ResNet-50 backbone integrated with a Feature Pyramid Network (FPN), as illustrated in Figure 5. The ResNet-50 backbone was used to extract hierarchical image features, while the FPN enabled multi-scale feature representation for cervical cells of different sizes. Candidate cell regions were generated by the Region Proposal Network (RPN), followed by RoI Align and the classification, bounding-box regression, and mask prediction heads.

Standard Mask R-CNN implementations commonly use generic anchor scales that were originally designed for natural-image object detection. These default anchor configurations may be suboptimal for cervical cytology images because many diagnostically relevant nuclei and cell regions are small, densely distributed, and partially overlapping. As a result, generic anchors may fail to generate sufficient high-overlap proposals for small cervical-cell instances, leading to missed detections and reduced APs performance.

To address this limitation, the RPN anchor configuration was modified using biologically informed anchor scales of (16, 32, 64, 128, 256). The inclusion of smaller anchors, particularly 16 and 32 pixels, was intended to better match the size distribution of cervical-cell instances in the annotated dataset. Small instances were defined according to the COCO small-object convention as mask areas < 32^2^ pixels after preprocessing and resizing. This optimized anchor configuration improved the ability of the RPN to localize small nuclei and overlapping cell regions while maintaining detection capacity for medium and large cell instances.

### 3.5. Soft Calibration-Based Mask Refinement

After instance mask prediction, a soft calibration-based post-processing step was applied to refine the predicted cervical-cell masks while preserving the original instance structure generated by Mask R-CNN. Unlike hard segmentation strategies such as Watershed, which attempt to split connected regions into new fragments, the proposed soft calibration operates on each predicted instance mask independently. Its purpose is not to create new instances, but to remove small artifacts, smooth irregular boundaries, and improve morphological consistency.

For each predicted instance i, Mask R-CNN generates a probability mask (P_i(X,Y)). The predicted probability mask was converted into a binary mask using a fixed threshold (Equations (1) and (2)) τ = 0.5:

(1) M_i(X,Y) =1, if P_i(X,Y)≥τ 

(2) M_i(X,Y)=0, if P_i(X,Y)<τ 

After binary thresholding, small disconnected components with an area smaller than A_min=100 pixels were removed to suppress noise-induced false mask fragments. The remaining mask was then refined using the soft calibration procedure described in Equation (3):

(3) M_i^soft =Open_O(Close_C(RemoveSmall(M_i,A_min)))

where (C = 3) denotes the radius of the structuring element used for morphological closing, and (O = 2) denotes the radius of the structuring element used for morphological opening. Closing was applied first to fill small gaps and improve mask continuity, while opening was subsequently applied to remove thin artifacts and boundary irregularities.

The values (C = 3) and (O = 2) were selected empirically to provide mild boundary correction without excessive deformation of the predicted cervical-cell masks. A stronger calibration could remove fine cellular details or merge neighboring structures, whereas weaker calibration had limited effect on boundary artifacts. Therefore, the selected values provided a balance between mask smoothness and morphological preservation.

**Figure 6:**
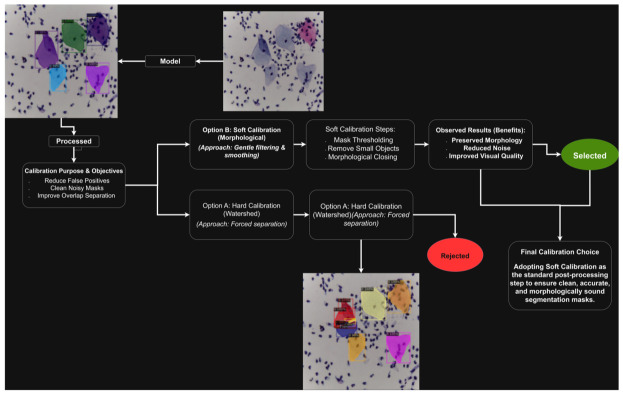
Soft calibration-based mask refinement workflow. The predicted Mask R-CNN probability mask is thresholded, small disconnected components are removed, and mild closing and opening operations are applied using (C = 3) and (O = 2), respectively. Unlike Watershed-based hard calibration, the proposed soft calibration preserves the original instance identity and avoids aggressive over-fragmentation.

This strategy differs from Watershed-based hard calibration because it does not impose a new segmentation topology or split predicted objects using distance-transform markers. Instead, it preserves the instance-level predictions generated by Mask R-CNN and applies only local morphological refinement. This distinction is important in dense Pap smear images, where aggressive hard splitting may over-fragment overlapping cells and reduce segmentation accuracy. The detailed steps of the proposed soft calibration-based mask refinement are presented in Algorithm 1. The overall workflow of the proposed soft calibration-based mask refinement is illustrated in Figure 6.
**Algorithm 1.** Soft calibration-based mask refinement.Input: Predicted probability masks (P_i), confidence scores (S_i), threshold (τ$\;=\;0.5),\;\mathrm{minimum}\;\mathrm{area}\;(A_{min}=100$), closing radius (C = 3), opening radius (O = 2). Output: Refined instance masks (M_i^soft). For each predicted instance mask (P_i), convert the probability mask into a binary mask using (τ = 0.5). $\mathrm{Remove}\;\mathrm{disconnected}\;\mathrm{components}\;\mathrm{smaller}\;\mathrm{than}\;(A_{min}=100$) pixels. Apply morphological closing using a disk-shaped structuring element with radius (C = 3). Apply morphological opening using a disk-shaped structuring element with radius (O = 2). Preserve the original instance score and instance identity. Return the refined mask (M_i^soft).

### 3.6. Evaluation Metrics and Clinical Cell-Count Assessment

The proposed Mask R-CNN framework was evaluated using two complementary assessment strategies: computational instance-segmentation evaluation on the annotated internal testing subset and clinical cell-count agreement assessment on the independent KAUH held-out dataset. This separation was necessary because the annotated public testing subset included pixel-level instance masks, whereas the KAUH external held-out dataset did not include pixel-level mask annotations.

For computational evaluation, standard COCO-style instance segmentation metrics were used. AP50 was used as the primary detection metric and represents the average precision at an intersection-over-union threshold of 0.50. AP75 was also reported to assess higher-precision localization. Mask IoU was used to measure the spatial overlap between predicted masks and ground-truth instance masks. In addition, size-specific average precision values were reported as APs, APm, and APl for small, medium, and large cervical-cell instances, respectively. Small instances were defined according to the COCO small-object convention as mask areas < 32^2^ pixels after preprocessing and resizing, medium instances as 32^2^–96^2^ pixels, and large instances as ≥96^2^ pixels. Inference time was recorded as seconds per image to evaluate computational efficiency.

The independent KAUH held-out dataset of 210 images was used for clinical cell-count agreement assessment rather than pixel-level segmentation metrics. For each KAUH image, five expert raters independently counted the visible cervical cells. The raters were blinded to the model outputs during the counting process, and model predictions were compared with the physician-derived reference counts only after all expert counts had been completed. The physician-derived reference count for image (i) was calculated as the arithmetic mean of the five raters’ counts, as shown in Equation (4).

(4) $$DoctorsMean_{i}=\frac{D1i+D2i+D3i+D4i+D5i}{5}$$

The absolute percentage error for each image was calculated by comparing the model-derived cell count with the physician-derived mean count, as defined in Equation (5).

(5) $$Erorr_{i}=\frac{\left|ModelCount_{i}-DoctorsMean_{i}\right|}{DoctorsMean_{i}}$$

The overall clinical detection accuracy was then calculated as 100% minus the Mean Absolute Percentage Error, as expressed in Equation (6).

(6) OverallAccuracy=100%−MeanAbsolutePercentageError

Using this approach, the overall clinical detection accuracy across the 210-image KAUH held-out dataset was 93.00%. This value represents agreement between the model-derived cell counts and the expert-derived reference counts. Therefore, it should be interpreted as clinical detection accuracy rather than diagnostic classification accuracy. A summary of the evaluation protocol, datasets, reference standards, reported metrics, and their corresponding purposes is presented in Table 3.

The detailed density-stratified clinical cell-count results are reported in Section 4.4.

## 4. Results

### 4.1. Qualitative Impact of Anchor Optimization

**Figure 7:**
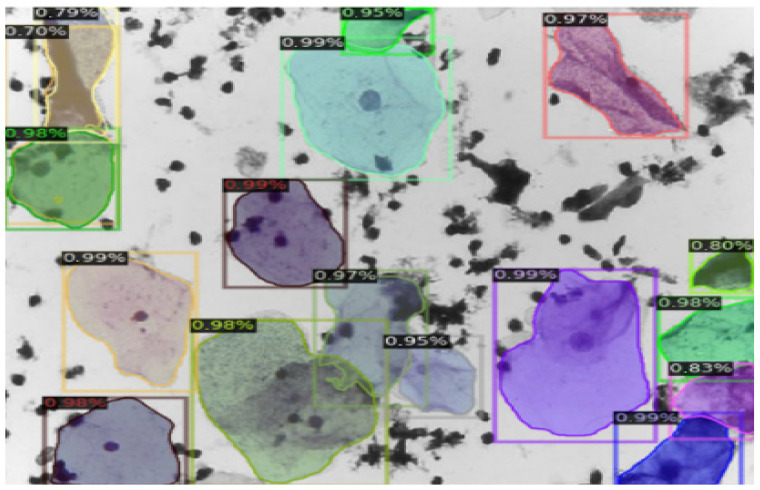
Representative segmentation output before structural optimization. The unoptimized configuration shows incomplete separation of adjacent cervical cells and reduced mask alignment in overlapping regions.

Figure 7 presents a representative segmentation output before applying the structural optimization of the Mask R-CNN framework. The unoptimized configuration showed incomplete separation of adjacent cervical cells, weak mask-to-cell correspondence, and reduced boundary alignment in overlapping regions. These limitations were more apparent in dense cellular regions, where small and partially overlapping cells were frequently missed or merged.

**Figure 8:**
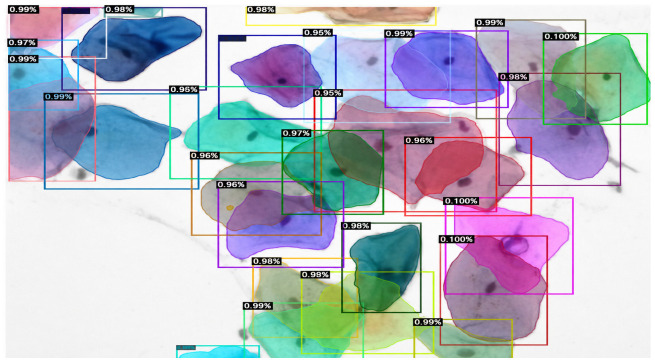
Representative segmentation output after anchor optimization. The modified anchor configuration improves proposal generation for small and overlapping cervical-cell instances, resulting in clearer cell separation and improved mask-to-cell correspondence. The numbers above the bounding boxes indicate the confidence scores assigned by the model to each detected cervical-cell instance.

After applying the optimized anchor configuration, the model showed improved proposal generation for small and overlapping cervical-cell instances, as illustrated in Figure 8. The modified Region Proposal Network using anchor scales (16, 32, 64, 128, 256) improved the ability of the model to localize small nuclei and overlapping cell regions. Compared with the unoptimized output, the optimized model produced clearer cell separation, improved mask alignment, and better preservation of cellular morphology across different cell sizes.

### 4.2. Final Quantitative Performance

The final optimized Mask R-CNN framework with biologically informed anchor scales and soft calibration achieved strong instance segmentation performance on the original evaluation images. The proposed framework reached an AP50 of 89.90% and a Mask IoU of 90.44%. Size-specific evaluation showed an APs of 22.80% for small cervical-cell instances, an APm of 68.45% for medium instances, and an APl of 81.42% for large instances. These results indicate that the proposed framework maintained high overall detection accuracy while improving mask-level spatial agreement and small-cell detection performance. The detailed quantitative evaluation results are summarized in Table 4.

**Figure 9:**
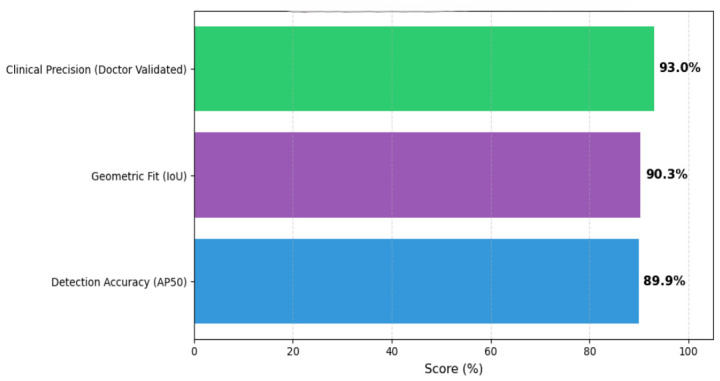
Final quantitative and clinical evaluation summary. The proposed framework achieved an AP50 of 89.90%, Mask IoU of 90.44%, APs of 22.80%, and clinical detection accuracy of 93.00% on the independent KAUH held-out assessment.

The APs value was reported explicitly to clarify small-cell performance, as small cervical-cell instances were defined using the COCO small-object convention as mask areas < 32^2^ pixels after preprocessing and resizing. The lower APs compared with APm and APl indicates that small-cell detection remains more challenging; however, the optimized anchor configuration improved sensitivity to small cervical-cell structures. Representative examples of the optimized segmentation performance are presented in Figure 9.

### 4.3. Component-Wise Ablation Analysis

A component-wise ablation analysis was conducted to evaluate the contribution of the main framework components, including the standard baseline Mask R-CNN model, soft calibration alone, optimized anchor configuration, hard calibration, and the full proposed framework. The baseline model used the same preprocessing and on-the-fly augmentation pipeline but retained the default Mask R-CNN anchor configuration and did not include soft calibration.

The baseline Mask R-CNN model achieved limited segmentation performance, with an AP50 of 10.04% and an APs of 0.997%. Applying soft calibration alone to the baseline model did not improve performance and reduced AP50 to 2.73%, indicating that post-processing cannot compensate for inadequate proposal generation when default anchors fail to detect small cervical-cell instances. In contrast, introducing biologically informed anchor scales (16, 32, 64, 128, 256) substantially improved performance, increasing AP50 to 89.90% and APs to 22.45%. The full proposed framework, which combined optimized anchors with soft calibration, preserved AP50 at 89.90% while improving APs to 22.80%, APm to 68.45%, and APl to 81.42%. The quantitative comparison of the baseline model and each framework component is presented in Table 5.

**Figure 10:**
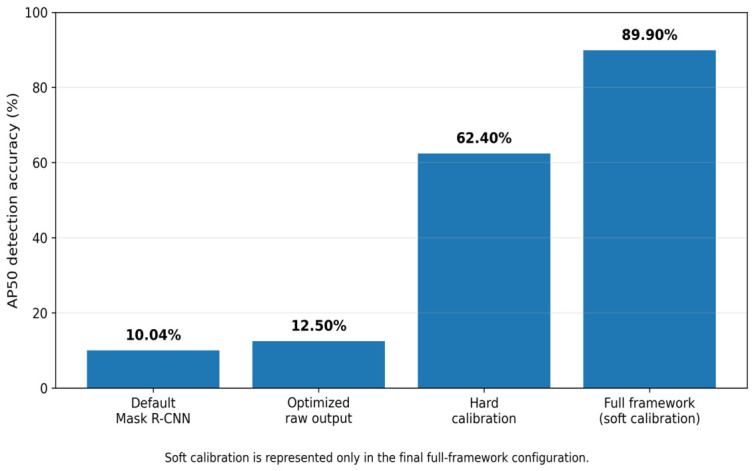
Component-wise ablation comparison of the proposed framework. The analysis compares the baseline Mask R-CNN model, baseline with soft calibration only, optimized anchor output, hard calibration, and the full proposed framework.

**Figure 11:**
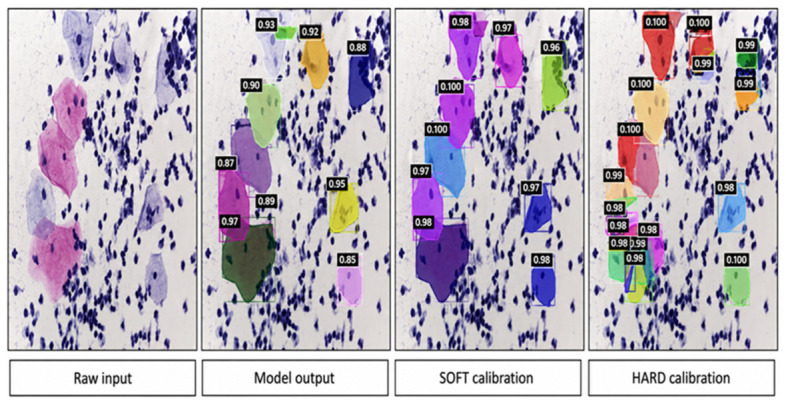
Visual comparison between hard and soft calibration. Hard calibration introduces over-fragmentation and excessive splitting, whereas soft calibration refines mask boundaries while preserving instance identity and cellular morphology. The different colors are used only for visual distinction between segmented cell instances and do not represent different cell types, classes, or biological structures.

These results indicate that anchor optimization was the main contributor to improved small-cell detection, while soft calibration provided additional mask refinement only after reliable instance proposals had been generated. The poor performance of Watershed-based hard calibration further confirms that aggressive splitting is unsuitable for dense and overlapping Pap smear images because it introduces excessive fragmentation and reduces mask-level accuracy. A quantitative comparison of the component-wise ablation results is presented in Figure 10. A visual comparison between hard and soft calibration is presented in Figure 11, highlighting the advantages of soft calibration in preserving cell morphology while reducing over-fragmentation.

### 4.4. Clinical Cell-Count Agreement on the KAUH Held-Out Dataset

Clinical cell-count agreement was evaluated on the independent 210-image KAUH held-out dataset. This dataset was not used for model training, validation, or internal testing. Because pixel-level mask annotations were not available for the KAUH images, this evaluation focused on agreement between model-derived cell counts and physician-derived reference counts rather than AP50 or Mask IoU.

The overall clinical detection accuracy was 93.00% using the mean count of five blinded expert raters as the reference standard. Accuracy was highest in low-density images and decreased gradually as cell density increased, indicating that dense overlapping regions remain more challenging for automated segmentation. The detailed clinical cell-count agreement results across the different cell-density categories are summarized in Table 6.

The weighted overall clinical detection accuracy was calculated according to the number of images in each density group, as shown in Equation (7):

(7) $$OverallAccuracy=\frac{\left(70\times 95.00\%\right)+\left(90\times 93.00\%\right)+\left(50\times 90.20\%\right)}{210}=93.00\%$$

The decrease in clinical detection accuracy from low-density to high-density images indicates that dense cellular overlap remains more challenging for automated instance segmentation. Nevertheless, the overall agreement of 93.00% supports the potential utility of the proposed framework as an auxiliary cervical-cell detection and segmentation tool.

### 4.5. Final Segmentation Outputs

**Figure 12:**
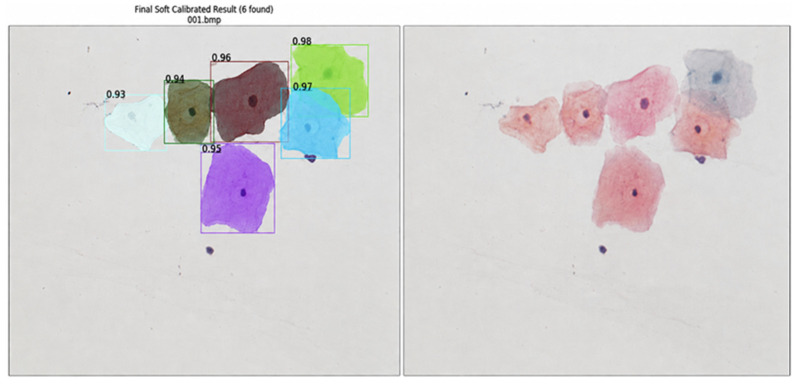
Final segmentation output after anchor optimization and soft calibration, showing improved separation of overlapping cervical-cell instances. Different colors are used only to distinguish individual segmented cell instances for visualization purposes and do not indicate different cell types or categories.

**Figure 13:**
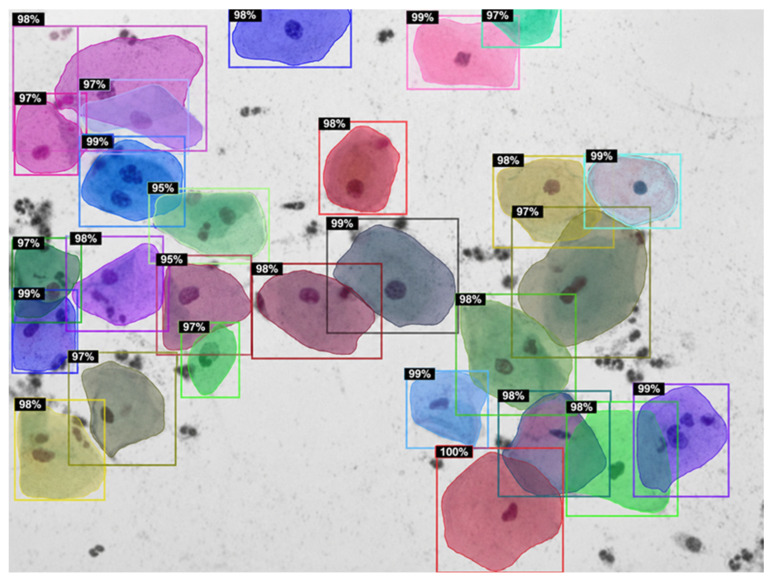
Final segmentation output demonstrating refined mask boundaries and improved preservation of cellular morphology after soft calibration. Different colors are used only to distinguish individual segmented cell instances for visualization purposes and do not indicate different cell types or categories.

**Figure 14:**
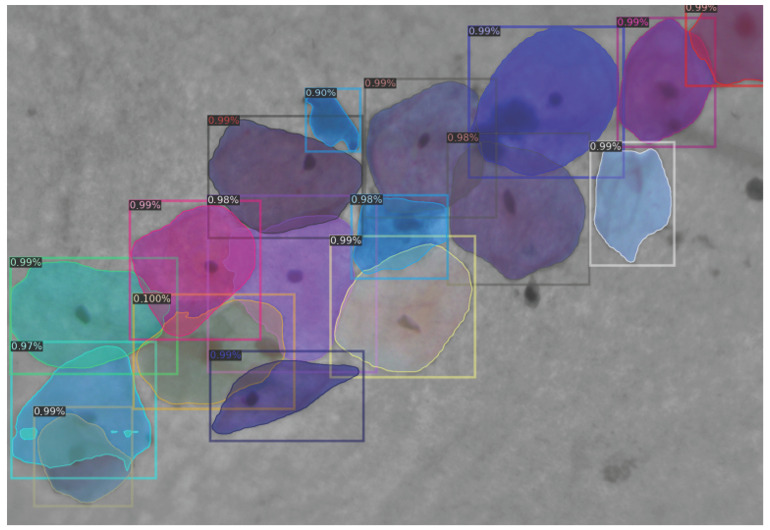
Final segmentation output on an unseen test image, illustrating the ability of the proposed framework to detect and segment dense cervical-cell regions. Different colors are used only to distinguish individual segmented cell instances for visualization purposes and do not indicate different cell types or categories.

Representative qualitative outputs are shown in Figure 12, Figure 13 and Figure 14. The final model outputs demonstrate improved separation of overlapping cervical cells, clearer cell-to-mask correspondence, and improved preservation of cellular morphology after applying the optimized anchor configuration and soft calibration. The final masks show better alignment with visible cell boundaries compared with the uncalibrated output, particularly in regions containing dense or partially overlapping cells.

### 4.6. Comparison with Existing Methods

To further contextualize the performance of the proposed framework, a comparative analysis was conducted against representative cervical-cell segmentation approaches reported in the literature. These methods include level-set-based segmentation [2,3], Watershed-based and classical segmentation approaches [4], prior-based variational models [5], deep convolutional segmentation methods [17], IRNet-based instance segmentation [19], Mask R-CNN-based approaches [24,25], PointRend-enhanced refinement [20], transformer-based Mask2Former segmentation [26], and recent deep learning-enabled liquid-based cervical cytology models for cervical precancer and cancer detection [27].

It is important to note that the comparison should be interpreted as a methodological and metric-level comparison rather than a fully controlled head-to-head benchmark. This is because previously published studies commonly differ in dataset source, annotation protocol, image resolution, preprocessing pipeline, evaluation strategy, and reported metrics. Nevertheless, this comparison is useful for positioning the proposed framework relative to traditional segmentation, deep instance segmentation, boundary-refinement, and transformer-based approaches.

Traditional segmentation methods, such as level-set models [2,3], Watershed-based approaches [4], and prior-based variational models [5], have been widely used for cervical-cell and nucleus segmentation. These approaches can be effective when cell boundaries are clear and cells are relatively separated. However, their performance is often limited under staining variation, illumination inconsistency, weak boundaries, and dense cellular overlap. In particular, Watershed-based segmentation may lead to over-fragmentation when applied to clustered cervical cells, which is consistent with the degraded performance observed in the hard calibration experiment in this study.

Deep learning methods, including deep convolutional segmentation [17], IRNet [19], standard Mask R-CNN [24], improved Mask R-CNN [25], PointRend-enhanced Mask R-CNN [20], and transformer-based Mask2Former [26], have improved segmentation robustness by learning hierarchical and contextual image representations. More recently, deep-learning approaches have also been extended to cervical cytology nuclei segmentation in whole-slide images, demonstrating the feasibility of AI-assisted analysis for large-scale cervical cytology assessment [28]. However, general-purpose detection and segmentation architectures may still be limited when small cervical nuclei and dense overlapping cell regions are present. In particular, generic anchor configurations may fail to generate sufficient high-overlap proposals for small cervical-cell instances, reducing small-cell sensitivity.

The proposed framework addresses these limitations by combining biologically informed anchor scales (16, 32, 64, 128, 256) with soft calibration-based mask refinement. The optimized anchor configuration improves proposal generation for small and overlapping cervical-cell instances, while soft calibration refines predicted masks without imposing aggressive object splitting. As a result, the proposed framework achieved an AP50 of 89.90%, a Mask IoU of 90.44%, an APs of 22.80%, an APm of 68.45%, and an APl of 81.42%. A comparative summary of the proposed framework and representative cervical-cell segmentation methods is presented in Table 7. 

Overall, the proposed framework showed advantages in three main aspects. First, the optimized anchor configuration improved the proposal stage for small cervical-cell instances, directly addressing the low small-cell sensitivity associated with generic anchor scales. Second, the soft calibration strategy refined mask boundaries while preserving instance identity, unlike Watershed-based hard calibration, which caused excessive over-fragmentation. Third, the framework maintained strong performance across small, medium, and large cell instances, supporting its suitability for dense and overlapping Pap smear images. However, because many prior studies used different datasets and evaluation protocols, future work should include a fully controlled benchmark in which representative SOTA methods are reimplemented and evaluated on the same annotated cervical cytology test set.

## 5. Discussion

This study highlights several important considerations for interpreting the performance of the proposed optimized Mask R-CNN framework for cervical-cell instance segmentation. The final framework combined a ResNet-50-FPN-based Mask R-CNN architecture, biologically informed anchor optimization, and soft calibration-based mask refinement. The proposed method achieved an AP50 of 89.90%, a Mask IoU of 90.44%, and size-specific AP values of 22.80%, 68.45%, and 81.42% for small, medium, and large cervical-cell instances, respectively. In addition, clinical cell-count agreement on the independent 210-image KAUH held-out dataset reached 93.00% when the mean count of five blinded expert raters was used as the reference standard.

The dataset used in this study remains relatively limited for deep learning-based instance segmentation. Although the public development image pool included 460 Pap smear images, only the annotated COCO-format subset was used for pixel-level computational training and mask-based evaluation. In addition, the images were assembled from different sources, which introduced variability in contrast, brightness, staining intensity, fixation protocols, and acquisition conditions. Such heterogeneity may increase exposure to diverse imaging patterns, but it can also introduce domain-specific bias and affect generalizability. Differences between conventional smear preparations and liquid-based cytology may further contribute to variations in cellular morphology and illumination. Therefore, future studies should focus on larger, standardized, multi-center datasets to ensure robust validation across different clinical environments. This need is consistent with recent cervical cytology resources and large-scale studies, including annotated cervical cytology datasets for AI benchmarking, liquid-based cytology models for cervical precancer and cancer detection, and whole-slide-image segmentation studies that emphasize the effect of acquisition variability and institutional data differences on model performance [27,28,29].

The proposed framework primarily addresses instance segmentation by identifying and delineating individual cervical cells within complex and overlapping clusters. This directly supports cell localization, boundary definition, and cell-count estimation. However, segmentation alone does not complete the diagnostic workflow. This is consistent with recent evidence showing that single-cell segmentation can be combined with downstream deep learning classification to improve automated cervical cancer screening, particularly when liquid-based cytology images are used [30]. For full clinical applicability, future work should incorporate a classification module capable of categorizing segmented cells according to established reporting systems such as the Bethesda classification. Such integration would shift the framework from a cell-detection and segmentation tool toward a more comprehensive diagnostic support system.

A key finding of this study is that structural adaptation of the Mask R-CNN proposal stage is important for cervical cytology images. Standard Mask R-CNN configurations commonly use generic anchor scales designed for natural-image object detection. These anchors may be poorly matched to small cervical nuclei and dense overlapping cellular structures. Similar challenges have been reported in previous studies, where general-purpose segmentation models struggled to capture morphological variability and scale differences in cervical-cell images [24,25]. In this study, the Region Proposal Network was adapted using anchor scales (16, 32, 64, 128, 256), which better matched the size distribution of cervical-cell instances and improved proposal generation for small and partially overlapping cells.

The explicit reporting of APs further clarified the impact of anchor optimization on small-cell performance. Small cervical-cell instances were defined according to the COCO small-object convention as mask areas < 32^2^ pixels after preprocessing and resizing. The final APs of 22.80% indicates improved sensitivity to small cervical-cell structures, although small-cell detection remained more difficult than medium- and large-cell detection. This finding is expected because small nuclei provide fewer pixels for feature extraction and are more likely to be affected by overlap, staining variation, and local artifacts. These observations are consistent with prior work emphasizing the importance of task-specific architectural adaptation in medical image segmentation [17,19].

The post-processing analysis also demonstrated that the choice of calibration strategy has a substantial effect on segmentation quality. Classical techniques such as Watershed-based algorithms can be sensitive to noise and may perform poorly in dense cellular clusters [2,4]. In this study, Watershed-based hard calibration substantially reduced performance, with AP50 decreasing to 5.08% and Mask IoU to 49.19%. This degradation suggests that aggressive splitting may produce excessive fragmentation when cell boundaries are weak or when cytoplasmic regions overlap. In contrast, the proposed soft calibration preserved the original instance identity and improved mask refinement without imposing a new segmentation topology. Soft calibration maintained AP50 at 89.90% while improving Mask IoU from 90.30% to 90.44% and APs from 22.45% to 22.80%.

When compared with existing approaches, the proposed method provides a balanced solution for overlapping cervical-cell segmentation. Classical segmentation methods, including level-set and Watershed-based algorithms, are often limited by staining variation, weak boundaries, and dense overlap [2,4]. CNN-based models have improved segmentation accuracy but may still encounter difficulties in severe cytoplasmic overlap and nuclear clustering [13,17]. Mask R-CNN-based frameworks have shown promise for separating overlapping objects [24,25], while transformer-based segmentation frameworks, such as Mask2Former with denoising strategies, reflect growing interest in boundary-aware and context-aware models for dense cytological scenes [26]. In this context, the proposed optimized Mask R-CNN framework combines task-specific anchor adaptation with soft calibration to improve small-cell detection, preserve cellular morphology, and reduce over-fragmentation.

The clinical cell-count evaluation provides a practical perspective beyond computational mask-level metrics. Because the KAUH external held-out dataset did not include pixel-level instance masks, it was not used for AP50, APs, APm, APl, or Mask IoU computation. Instead, model-derived cell counts were compared with physician-derived reference counts calculated as the mean count of five blinded expert raters. The overall clinical detection accuracy of 93.00% indicates strong agreement between the model and expert-based cell-count assessment. However, this result should be interpreted as clinical detection accuracy, not diagnostic classification accuracy. The framework was evaluated for cell detection, segmentation, and count agreement rather than for assigning diagnostic categories or replacing cytopathology interpretation.

Overall, the proposed framework demonstrates that task-specific optimization of Mask R-CNN can improve cervical-cell instance segmentation in challenging Pap smear images. The combination of smaller biologically informed anchors and soft calibration provides a practical strategy for improving small-cell detection and mask refinement while avoiding the over-fragmentation associated with hard post-processing methods. Nevertheless, larger multi-center validation, broader external testing, and direct benchmarking against representative SOTA methods under identical experimental settings are still required before routine clinical deployment.

## 6. Limitations

Despite the promising results, several limitations should be acknowledged. First, although the public development image pool included 460 Pap smear images, only the annotated COCO-format subset was available for pixel-level computational training and mask-based evaluation. This remains relatively limited for deep learning-based medical image segmentation. Dynamic on-the-fly augmentation was applied to increase visual variability and reduce overfitting; however, augmentation cannot fully replace the richness, variability, and biological diversity of larger real-world clinical datasets.

Second, the independent KAUH held-out dataset consisted of 210 images collected from a single hospital in Jordan. Although this dataset provided an external clinical/inference assessment, it may still reflect institution-specific characteristics related to staining protocols, smear preparation, imaging devices, acquisition conditions, and patient population. Therefore, the generalizability of the model to other hospitals, laboratories, scanners, and staining protocols remains to be confirmed through larger multi-center validation studies.

Third, the KAUH external held-out dataset did not include pixel-level instance mask annotations. For this reason, AP50, APs, APm, APl, and Mask IoU could not be computed on the KAUH dataset. Instead, the KAUH assessment was based on clinical cell-count agreement between the model-derived counts and the physician-derived reference counts. Therefore, the reported 93.00% value should be interpreted as clinical detection accuracy based on cell-count agreement, not as diagnostic classification accuracy.

Fourth, the proposed framework currently focuses on instance segmentation and cell-count assessment. It does not include a downstream pathological classification module for assigning diagnostic categories such as NILM, LSIL, HSIL, or SCC. Although accurate segmentation is an important step in the cervical cytology workflow, full diagnostic support would require integration with a validated classification component and assessment against established reporting systems such as the Bethesda system.

Finally, the comparison with existing methods was limited by differences in datasets, annotation protocols, image resolutions, preprocessing pipelines, and reported evaluation metrics across published studies. Therefore, the SOTA comparison should be interpreted as a methodological and metric-level comparison rather than a fully controlled head-to-head benchmark. Future work should include reimplementation and evaluation of representative methods on the same annotated test set to provide a more rigorous comparison.

## 7. Conclusions

This study introduced an optimized deep learning framework for cervical-cell instance segmentation in Pap smear images, with particular emphasis on small-cell detection and the separation of overlapping cellular regions. The proposed framework was based on Mask R-CNN with a ResNet-50-FPN backbone and incorporated biologically informed anchor optimization together with soft calibration-based mask refinement.

The final framework achieved an AP50 of 89.90%, a Mask IoU of 90.44%, and size-specific AP values of 22.80%, 68.45%, and 81.42% for small, medium, and large cervical-cell instances, respectively. These findings demonstrate that the proposed anchor configuration improved the model’s ability to detect small and overlapping cervical-cell structures while maintaining strong overall segmentation performance.

The clinical cell-count assessment on the independent 210-image KAUH held-out dataset achieved a clinical detection accuracy of 93.00% when compared with the mean count of five blinded expert raters. This result supports the potential value of the framework as an auxiliary cell-detection and segmentation tool for cytopathology workflows. However, this value should be interpreted as cell-count agreement rather than diagnostic classification accuracy.

Overall, the findings suggest that combining biologically informed anchor scales with soft calibration can improve cervical-cell instance segmentation in challenging Pap smear images. Nevertheless, further validation using larger multi-center datasets, pixel-level external annotations, and downstream pathological classification modules is required before routine clinical deployment.

## 8. Future Work

Future work should focus on extending the proposed segmentation framework toward a more comprehensive cervical cytology decision-support system. While the current model addresses the question of where individual cervical cells are located by performing instance segmentation and cell-count assessment, the next step is to address the question of what type of cell or abnormality is present.

One important direction is the development of a downstream classification module that uses the segmented single-cell regions produced by the proposed framework. This module could be based on convolutional neural networks, transformer-based architectures, or classical machine learning approaches applied to extracted morphological and textural features. The goal would be to classify segmented cells according to clinically relevant cervical cytology categories, such as NILM, LSIL, HSIL, and SCC, and to align the output with established reporting systems such as the Bethesda system.

Another priority is the expansion of the dataset. Future studies should include larger multi-center datasets with pixel-level instance annotations from different hospitals, laboratories, scanners, staining protocols, and slide-preparation methods. This would allow more rigorous evaluation of model generalizability and domain robustness. In particular, external datasets should include pixel-level mask annotations so that AP50, APs, APm, APl, and Mask IoU can be computed across centers rather than relying only on cell-count agreement.

Future work should also include direct benchmarking against representative state-of-the-art methods under identical experimental conditions. Reimplementing and evaluating methods such as standard Mask R-CNN, IRNet, PointRend-enhanced models, and transformer-based segmentation models on the same annotated test set would provide a stronger and more controlled comparison.

Finally, future research should investigate nuclear-region analysis in greater detail. Because nuclear morphology is central to cervical cytology interpretation, accurate segmentation could be combined with nuclear feature extraction to analyze nuclear size, shape irregularity, chromatin texture, and nucleus-to-cytoplasm characteristics. These extensions would help transform the current segmentation-focused framework into a more complete and clinically meaningful diagnostic support system.

## Figures and Tables

**Table 1:** Summarizes the dataset components, annotation status, and evaluation role.

Dataset Component	Images	Annotation Status	Role in This Study	Training	Validation	Internal Testing	External Clinical Assessment	Metrics Computed
Annotated public development subset	346	Pixel-level COCO instance masks available	Computational training, validation, and internal testing	242	52	52	0	AP50, APs, APm, APl, Mask IoU
Additional public development images	114	Pixel-level masks not available	Image-level inspection and qualitative inference assessment only	0	0	0	0	Not used for mask-based metrics
Full public development image pool	460	Partially annotated	Overall public development image pool	—	—	—	—	Mask metrics computed only on the annotated subset
KAUH external held-out dataset	210	Pixel-level masks not available	External clinical cell-count agreement assessment	0	0	0	210	Clinical detection accuracy based on cell-count agreement

**Table 2:** Cell-instance size distribution in the annotated COCO subset.

Split	Images	Annotated Cell Instances	Small Instances, Area < 32^2^ px	Medium Instances, 32^2^–96^2^ px	Large Instances, Area ≥ 96^2^ px	Small-Instance Proportion (%)	Median Mask Area (px^2^)	Mean Mask Area (px^2^)
Training	242	6311	473	3364	2474	7.49	6961.72	8464.02
Validation	52	1222	88	643	491	7.20	6997.66	8387.90
Internal testing	52	1327	89	670	568	6.71	7651.56	8935.53

**Table 3:** Evaluation protocol and metric usage.

Evaluation Component	Dataset Used	Number of Images	Reference Standard	Metrics Reported	Purpose
Computational instance-segmentation evaluation	Annotated internal testing subset	52	Pixel-level COCO-format instance masks	AP50, AP75, APs, APm, APl, Mask IoU, inference time	To quantify mask-based detection and segmentation performance
Clinical cell-count agreement assessment	Independent KAUH external held-out dataset	210	Mean count of five blinded expert raters	MAPE and clinical detection accuracy	To assess agreement between model-derived cell counts and expert-derived reference counts

**Table 4:** Final quantitative performance of the proposed framework.

Metric	Value (%)
AP50	89.90
Mask IoU	90.44
Aps	22.80
APm	68.45
Apl	81.42

**Table 5:** Component-wise ablation analysis of the proposed framework.

Configuration	Anchor Configuration	Post-Processing	AP50 (%)	AP75 (%)	APs (%)	APm (%)	APl (%)	Inference Time
Baseline Mask R-CNN model	Default Mask R-CNN anchors	None	10.04	4.58	0.997	3.23	9.94	0.2402 s/image
Baseline Mask R-CNN + soft calibration only	Default Mask R-CNN anchors	Soft calibration (C = 3, O = 2)	2.73	0.77	0.29	0.44	2.12	0.3538 s/image
Optimized Mask R-CNN output	(16, 32, 64, 128, 256)	None	89.90	—	22.45	68.12	81.35	0.1577 s/image
Optimized Mask R-CNN + hard calibration	(16, 32, 64, 128, 256)	Watershed-based hard calibration	5.08	—	0.03	2.90	16.67	1.3587 s/image
Full proposed framework	(16, 32, 64, 128, 256)	Soft calibration (C = 3, O = 2)	89.90	—	22.80	68.45	81.42	0.9147 s/image

**Table 6:** Clinical cell-count agreement on the independent KAUH held-out dataset.

Image-Density Group	Number of Images	Mean Physician Count ± SD	Mean Model Count	MAPE (%)	Clinical Detection Accuracy (%)
Low density	70	15.4 ± 2.1	14.8	5.00	95.00
Medium density	90	42.5 ± 4.3	44.2	7.00	93.00
High density	50	88.1 ± 8.7	81.5	9.80	90.20
Overall weighted average	210	—	—	7.00	93.00

**Table 7:** Comparison with representative cervical-cell segmentation methods.

Method Category	Representative Method	Main Strategy	Strengths	Main Limitations	Reported/Comparable Metrics
Traditional segmentation	Lu et al. [2,3]	Level-set-based segmentation of overlapping cervical cells	Useful for boundary-based segmentation and overlapping-cell analysis	Sensitive to initialization, weak boundaries, staining variation, and complex overlap	Commonly reports overlap-based metrics; AP50 usually not reported
Traditional segmentation	Chankong et al. [4]	Classical cervical-cell segmentation and classification	Simple and interpretable pipeline	Limited robustness under dense overlap, weak boundaries, and staining variability	Dice/accuracy-style metrics; not directly comparable to COCO AP
Prior-based segmentation	Nosrati and Hamarneh [5]	Variational segmentation with star-shape prior	Improves shape regularity for overlapping cells	Assumes prior morphology and may struggle with irregular cervical-cell shapes	Overlap-based metrics; AP50 usually not reported
Deep learning	Wan et al. [17]	Deep convolutional segmentation of overlapping cervical cells	Improved robustness compared with classical segmentation	Requires annotated data and may still struggle with small dense objects	Dataset-dependent; direct comparison limited
Instance segmentation	Zhou et al. [19]	IRNet-based overlapping cervical-cell segmentation	Designed for instance-level separation in overlapping cells	Requires careful training and dataset-specific optimization	Dataset-dependent; direct AP50 comparison limited
Instance segmentation	Chen and Zhang [24]	Mask R-CNN-based segmentation of overlapping cervical cells	Strong instance segmentation baseline	Generic anchor configuration may miss small nuclei and dense cell clusters	Dataset-dependent; AP50/IoU not always directly comparable
Improved Mask R-CNN	Wang et al. [25]	Improved Mask R-CNN for cervical-cell segmentation	Builds on an established instance segmentation framework	May still require dataset-specific tuning and validation	Dataset-dependent; direct comparison limited
Boundary refinement	Zhang et al. [20]	PointRend-enhanced cervical-cell segmentation	Improves fine boundary refinement	Does not directly solve small-object proposal mismatch	Dataset-dependent; AP50/IoU not always directly comparable
Transformer-based segmentation	Zhang et al. [26]	Mask2Former with denoising for overlapping cervical-cell segmentation	Strong global contextual modeling and flexible mask prediction	Computationally heavier and may require larger datasets	Dataset-dependent; direct comparison limited
Proposed framework	This study	Optimized Mask R-CNN with anchor scales (16, 32, 64, 128, 256) and soft calibration	Improves small-cell detection, preserves instance identity, and reduces over-fragmentation	Requires further validation on larger multi-center datasets	AP50 = 89.90%, Mask IoU = 90.44%, APs = 22.80%, APm = 68.45%, APl = 81.42%

## Data Availability

The data used in this study are available from the corresponding author. Upon reasonable request. The dataset is not publicly available due to privacy and ethical restrictions.

## Abbreviations

The following abbreviations are used in this manuscript:

AP	Average Precision
AP50	Average Precision at an Intersection-over-Union (IoU) threshold of 0.50
AP75	Average Precision at an Intersection-over-Union (IoU) threshold of 0.75
APS	Average Precision for small objects
CNN	Convolutional Neural Network
FPN	Feature Pyramid Network
IoU	Intersection over Union
RPN	Region Proposal Network
KAUH	King Abdullah University Hospital
NILM	Negative for Intraepithelial Lesion or Malignancy
LSIL	Low-grade Squamous Intraepithelial Lesion
HSIL	High-grade Squamous Intraepithelial Lesion
SCC	Squamous Cell Carcinoma
